# Health inequalities tackled through intersectoral collaboration: longitudinal process issues and insights

**DOI:** 10.1093/heapro/daaf234

**Published:** 2026-01-14

**Authors:** James Woodall, Paige Davies, Jenny Woodward, Susan Coan

**Affiliations:** School of Health, Leeds Beckett University, Portland Way, Leeds LS1 3HE, United Kingdom; School of Health, Leeds Beckett University, Portland Way, Leeds LS1 3HE, United Kingdom; School of Health, Leeds Beckett University, Portland Way, Leeds LS1 3HE, United Kingdom; School of Health, Leeds Beckett University, Portland Way, Leeds LS1 3HE, United Kingdom

**Keywords:** health inequalities, intersectoral collaboration, Ottawa Charter, community engagement, power dynamics, trust and governance, longitudinal qualitative research

## Abstract

This study contributes to ongoing reflections and debate on the legacy of the Ottawa Charter by illustrating how contemporary forms of intersectoral collaboration can be mobilized to address persistent health inequalities. Collaborations involving organizations from diverse sectors are often viewed as well-positioned to tackle complex health challenges, yet they frequently encounter political, organizational and cultural barriers that hinder their effectiveness. This paper uses a longitudinal approach to explore issues in relation to the formation and sustainability of a multi-sector collaboration in one geographic area in the UK, working under the banner of the Health Determinants Research Collaboration (HDRC)—a programme which seeks to further understand health determinants and to improve health outcomes in communities. Through qualitative interviews at two time points—12 months apart—with constituents of the collaboration, the data demonstrated a clear and shared vision for the collaboration and a neat ‘dovetailing’ of skill-sets related to community brokerage; academic rigour; and statutory legitimacy. While the collaboration under focus here was in its infancy, cultural, and practical tensions in ways of working; trust issues; pace of working; and philosophy were predicted to, and indeed did, emerge and required careful monitoring to ensure intended outcomes were not derailed.

Contribution to Health Promotion

- The paper shows how different sectors can work together to reduce health inequalities in local communities.
- Highlights the importance of trust, shared goals, and clear communication in successful partnerships.
- Explains how cultural differences between organizations can affect collaboration and outcomes.
- Offers insights into managing power dynamics and decision-making in multi-sector projects.
- Provides lessons for improving future health collaborations through long-term relationship building.

## Introduction

The Ottawa Charter made clear the necessity for co-ordinated action from a range of government and non-government organizations to improve health equity (WHO 1986), as complex health and social issues are rarely addressed sufficiently by a single organization, department or sector (Woodall and Cross 2021). Indeed, theoretically at least, individuals or sectors working alone will often achieve inferior outcomes to those working together (Jones and Barry 2011). Collaboration and partnerships are therefore often observed as a ‘panacea’ for tackling complex health and social problems (Armistead *et al*. 2007) and are relatively commonplace in the UK, USA, and elsewhere (Alderwick *et al*. 2021). The size, scale, and scope of partnerships designed to improve population health are heterogeneous and diverse (Willis *et al*. 2016) and some have devised a typology of partnerships—which show varying levels of engagement and interaction between organizations, including: networking; co-operation; co-ordination; coalition; and full collaboration (Boydell 2001)—to aid conceptual understandings.

The Ottawa Charter four decades ago redefined health as a resource for everyday life, not merely the absence of disease, highlighting a range of prerequisites for health such as: poverty; education; infrastructure and material resources; and housing (WHO 1986, Cross and Woodall 2024). The UK government has consistently recognized that health differs by social groups with policy discourse acknowledging that creating greater health equity is necessary (Fransham *et al*. 2023). Addressing the root causes of health inequalities is rarely found in ‘traditional’ health services—such as hospitals and primary care (Woodall and Morley 2024)—and instead requires work across various sectors and partners not always seen to have a traditional health remit [e.g. local government, transport, voluntary and community sector (VCS), academia] (WHO 1986). Given the multifaceted factors influencing health inequalities, collaborative ways of working across sectoral boundaries seems a useful diagnosis to reversing poor health outcomes in communities. Indeed, most health issues are cross-sectoral in nature and embrace multiple policy arenas, communities, organizations, and professional groups (Perkins *et al*. 2020). These ways of working draw heavily on the notion of intersectoral collaboration, defined as:

a recognised relationship between part or parts of different sectors of society which has been formed to take actions on an issue to achieve health outcomes or intermediate health outcomes in a way which is more effective, efficient or sustainable than might be achieved by the health sector acting alone. (WHO 1997 p. 3)

While not understating the benefits of intersectoral collaboration to improve health outcomes during the Covid-19 pandemic (South *et al*. 2024), evidence shows a concerning trend that intersectoral collaboration to improve health has offered, at best, mixed results (Judge and Bauld 2006). Indeed, some communities are not seeing improved health outcomes and, in some cases, inequalities between communities are growing in relation to life expectancy and morbidity (Marmot *et al*. 2020). The aspirations of the Ottawa Charter for health equity now face complex, evolving challenges that were unimaginable in the 1980s (Woodall and Freeman 2020). Outcome evaluations have proved useful in increasing understanding of what works to tackle health inequalities (Cross and Woodall 2024), but fewer studies have provided an operational blueprint for partnerships or provided longitudinal illumination of the processes underpinning success or failure in these collaborations seeking to reduce health inequalities (Evans and Killoran 2000, Willis *et al*. 2016, McGill *et al*. 2020). Which collaborations work, for whom and in what context is therefore largely absent (Alderwick *et al*. 2021). In addition where the literature has paid attention to the process issues relating to intersectoral collaboration for improved health, the focus has often been on health and social care partnerships (Alderwick *et al*. 2021) with less attention paid to partnerships addressing the social determinants of health (Smith *et al*. 2009).

A scoping review of international literature synthesized nine key components necessary for positive partnership processes in promoting health (Corbin *et al*. 2018). These included: aligned vision and goals between partner constituents; broad participation from a range of stakeholders; clear leadership; communication within the partnership and external messaging about the partnership; clear role structure and accountability; a balance of partner resources; creating a harmony between maintaining the partnership and ‘producing’ as a partnership; being mindful of the external context and how this can impact on the partnership; and evaluation of the partnership and its functions. Overlap with these nine components were also observed in a study which identified the criticality of a shared mission, common purpose, and aligned partner-interests enabling a greater likelihood of meaningful action (Holt and Aveling 2023).

Despite evidence showing the key ingredients for collaboration success, it also highlights why some collaborations fail to achieve their intended mission. In reality, partnerships are difficult and costly to establish and maintain (Wildridge *et al*. 2004, Smith *et al*. 2009). Partnerships can fail because of ‘deep-rooted political, organizational and cultural barriers’ (Evans and Killoran 2000, p. 126) they also usually have limited ways to measure and evaluate progress (Jones and Barry 2011), so objectively understanding why partnerships fail can be difficult to fully understand. Hubley *et al*. (2021) suggests that partnerships in health promotion face challenges because of differing concepts of health promotion; values; visions and aims; and issues whereby smaller organizations get marginalized or ‘pushed out’ by larger ones.

Within the UK, the Health Determinants Research Collaboration (HDRC) is a programme which seeks to increase research capacity and capability within local government to understand health determinants and to improve health outcomes in communities (Hampshaw *et al*. 2024). Local authorities occupy a pivotal position in efforts to reduce health inequalities. Beyond their statutory responsibilities for public health, they hold significant policy-making authority, budgetary control, and political accountability, which enables them to influence upstream determinants of health. The HDRC reflects sentiments of the Ottawa Charter where health is strongly positioned as being the responsibility of all sectors (including local authorities), not just healthcare (WHO 1986). This is not the first national attempt to address inequalities in the UK, with several policy initiatives and delivery mechanisms established to lessen poor health for communities (Evans and Killoran 2000). The HDRC programme supports thirty local authorities who work with academic partners; often VCS organizations; decision-makers; and citizens to better understand health influences and to create conditions that will lead to improved health outcomes (NIHR 2024).

This paper focuses on the early implementation of the HDRC programme in one geographical area as well as the collaborative relationship developed prior to receiving funding; during the bid writing phase; and during the first 2 years of implementation. This paper contributes to the ongoing dialogue around the Ottawa Charter’s enduring relevance, particularly in its call for intersectoral action to address the social determinants of health. The Ottawa Charter has long served as a foundational framework for health promotion practice. Yet, within the health promotion community, there remains a lack of consensus on whether the Charter should be updated to reflect contemporary challenges—such as the commercial determinants of health and short-term political agendas (Thomas *et al*. 2025)—or continue to stand as the enduring cornerstone of policy and practice (Woodall and Freeman 2020). Indeed some have argued that health promotion has lost focus and is ‘going in all directions’ (Lindström 2018, 97). In marking the Charter’s 40th anniversary, we reflect on how the HDRC initiative embodies key principles of health promotion through collaborative governance and strengthening communities.

There is a range of semantics related to collaboration and partnership working that will not be rehearsed here (Armistead *et al*. 2007); however, it is worth noting the collaboration in this study had clear contractual and financial stipulations bounding the actors that would encompass the following definition:

a cross-sector, inter-organizational group, working together under some form of recognized governance, towards common goals which would be extremely difficult, if not impossible, to achieve if tackled by any single organization. (Armistead et al. 2007, 212)

In order to advance further understanding of the issues related to partnership working for addressing the social determinants of health, the data collection informing the paper had several key objectives, these were:

1. To explore the initial expectations of constituents working in a multi-sector collaboration and how this developed longitudinally during the collaboration.
2. To identify the facilitating and enabling factors which lead to successful collaboration between the statutory sector, voluntary sector and academic partners in addressing health inequalities.
3. To explore cultural differences in working practices between collaborators and how these were managed and resolved over time.

## Materials and methods

A qualitative design was adopted to explore the processes and experiences underpinning intersectoral collaboration. Qualitative approaches are particularly suited to examining complex social phenomena and capturing the perspectives of diverse stakeholders in detail (Patton 2014). Qualitative approaches aligned to the objectives of the study by enabling elucidation of the mechanisms of success and failure within the partnership. To facilitate this, semi-structured interviews with key constituents of the partnership were used to gather data. This method enabled participants the opportunity to talk in detail and depth about their unique experiences in a confidential manner and allowed flexibility to probe emerging issues while maintaining consistency across participants (Adeoye-Olatunde and Olenik 2021). To enhance neutrality, interviews were conducted by researchers external to the collaboration, which helped reduce preconceived assumptions and encouraged candid responses. Reflexivity was maintained throughout the research process, with researchers critically reflecting on their positionality and potential influence on data interpretation, consistent with best practice in qualitative inquiry (Braun and Clarke 2024).

Sampling was critical to enable a broad-based understanding of the partnership, both in its inception (i.e. bid writing) and during implementation. Purposive sampling was used to gain in-depth understanding from participants best placed to provide insights into the programme (Patton 2014). It was critical to gain the perspectives of those working across the sectors in the programme. In Year 1, eleven participants were interviewed; in Year 2, thirteen participants were interviewed using a semi-structured schedule. Of these, eight participants were interviewed twice to capture changes over time. Five participants were interviewed only in Year 2 because they joined the collaboration later, while three were interviewed only in Year 1 as they left during early implementation. Ultimately, the decision to interview participants once or twice was based on their timing of involvement in the collaboration. Participants interviewed represented those working in local government (including those working in both public health functions and those in broader policy and strategy); the VCS; an elected political member; and academics from two partner institutions. The interview schedule had some consistency at the two time points and covered the expectations and experiences of working in a multi-sector collaboration; successes and challenges; the benefits and barriers of working across sectors; and how sustainability of the collaboration was, and can be, maintained.

All interviews were undertaken via MS Teams and transcriptions generated through the software were checked for accuracy and to aid familiarization. Two researchers independently coded an initial selection of transcripts to collaboratively develop a coding framework that could be consistently applied across the entire dataset—this included latent and semantic codes. Throughout the coding process, researchers remained reflexively aware of their positionality as academic professionals, distinct from those working within local authorities or the VCS. This awareness informed their interpretation of the data, acknowledging that their analytical lens was shaped by their institutional context and such acknowledgement of this is considered good practice (Braun and Clarke 2024). Codes were agreed and discrepancies identified and resolved through mutual discussion. Following an established approach to thematic analysis (Braun and Clarke 2013), the full dataset was systematically coded using the collaboratively developed framework. This process involved a constant engagement with the data, allowing for both semantic (explicit) and latent (underlying) meanings to be captured.

Once initial coding was complete, a structured process of thematic organization was undertaken. This involved clustering codes that shared conceptual or experiential similarities, thereby forming preliminary thematic categories. These categories were then critically reviewed to ensure they were internally coherent and externally distinct. Where codes were found to be too broad or lacking specificity, they were disaggregated into more precise sub-codes to better reflect the nuances within the data. This iterative refinement helped to ensure that the final themes were both analytically robust and grounded in participants’ accounts.

Themes were not only shaped by frequency or repetition but also by their relevance to the research questions and their capacity to offer meaningful interpretations of the data. Throughout this process, researchers maintained reflexive awareness of their positionality and the potential influence of their academic standpoint on theme construction and interpretation.

## Results

This section presents five thematic areas deriving from interview analysis. Verbatim quotations have been used for illustrative purposes, but anonymized to protect participants.

### Collaborative skills and complementarity

The strength that collaborative working could bring to tackling complex issues was a highly salient issue across the data set. The notion that collaboration brings strength by offering a more holistic skill-set and network to tackle issues relating to health inequalities was noted by the majority of those interviewed. Each constituent recognized the assets of their own organization, but also were frank about their weaknesses and deficits in addressing health inequalities. Those participants representing the local authority highlighted the strengths they bring as statutory providers and the responsibilities they have as an organization to address public health. Conversely, there was broad acknowledgement that the organization’s links with communities could be stronger and moreover the best way to address this was through VCS providers who had established and trusted relationships with a myriad of communities:

I think for me there's a credibility with the third sector that we [the local authority] wouldn't otherwise have. So, actually, if we want to talk to our residents and communities, the VCS are the people that do that day in, day out. (Participant 1, local authority, Yr1)

Despite the VCS being lauded for their expertise in working with and alongside communities, VCS respondents recognized their organizations’ limitations around generating, analysing, interpreting, and optimizing research evidence and sometimes having the political levers to make meaningful change. That said, this was compensated through academic and local authority partners being able to address this potential void:

I guess one of our strap lines at [VCS organisation], unofficially, is that we are good at what we do, but we can’t do everything, and we are only a part of the jigsaw. (Participant 4, VCS, Yr1)

In the early formation of the collaboration, participants expected to learn from each other and find ways to ‘dovetail’ to maximize impact. They further recognized where the strengths and weaknesses of the constituents of the collaboration could be overlaid to ensure competence and expertise in strategic and operational delivery:

I think the benefit that it brings is everybody can learn from each other. I am a big believer in people have expertise, knowledge, skills, and experience in their own fields, and I think by bringing people together from VCS, academia and local authorities that everybody learns something from each other. (Participant 3, local authority, Yr1)

As the collaboration matured in Year 2 though, the initial skills that were identified as strengths of the partners were reported to be undermined by occasional risk-averse approaches within the local authority. As an example, academics within the partnership often faced additional challenges to undertaking data gathering due to additional processes prescribed by the local authority. Their skills in research ethics and data management were not always recognized and sometimes suppressed:

It's not the individual officers’ faults; they're just they're kind of like conditioned to work in a certain way and often that is around that kind of risk aversion and that risk aversion can become a barrier to research if it's not managed effectively. (Participant 13, local authority, Yr2)

The local authority participants recognized their own approach to managing partnerships could be problematic, but many sought to resolve this despite the ingrained ways of working that had already been established:

I think there's probably some lessons for us to learn as officers in terms of, yes, we need to hold people to be accountable, but do we need to be a little bit more flexible and I don't know the answer to that. (Participant 3, local authority, Yr1)

### Power dynamics and decision-making

When the collaboration was conceived there were expectations, albeit implicit, that each partner was equal and that ‘power’ was equally distributed. It was assumed that the collaborative arrangement at the point of contracting the partnership would temper any domination by a single institution:

I'm hoping that there will be a genuine shift in the culture within statutory services whereby communities are genuinely partners and partners who have equal value in designing services and I'm hoping that the paternalistic culture that can be evident within statutory services will dissipate, and it will become less paternalistic. (Participant 5, VCS, Yr1)

As the collaboration began to deliver aspects of work towards the end of the first year, and started to make key decisions around marketing strategies and engagement with communities, it became apparent that the local authority held a greater actual share of power than others and were, in effect, the ultimate decision-makers. This raised questions and concerns about the nature of the partnership and stimulating reflection as to whether a true partnership could materialize. Arguably the local authority were the leading organization in the bidding process with financial accountability resting predominantly in this organization. One participant candidly reflected:

I think the problem is that actually the only real decision maker is in the council and whilst, like it's not that I disagree with them, I don't think you can have true partnership if you've only got one decision maker. (Participant 16, local authority, Yr2)

### Organizational memory

It was anticipated that a key element of potential success in the current collaboration was a previous track-record in working together and the organizational histories between constituent organizations. In this particular context, VCS colleagues had worked previously with the local authority and moreover the academic partners had previously had successful research bids and projects with the local authority also. These past relationships provided some levels of assurance at the bidding stage that future endeavours would be successful:

You know we have risen to the challenge and because we've worked collaboratively over the last four or five years, we're open to challenge and scrutiny…So, I think we work very well together in that regard. (Participant 7, local authority, Yr1)

Notwithstanding, there were also examples where the organizational past could act as barrier for effective collaborative working and that some historical issues may have a deleterious impact in the short-term establishment of the group:

Our VCS partners, they don't always like the council. They've got some very understandable gripes about the council, particularly when we come in and tell them what to do. (Participant 1, local authority, Yr1)

This materialized in Year 2 as mistrust was commented upon within the collaboration and a sense that partners were stifled by micro-management of the day-to-day elements of the work:

There definitely has felt at times…it's felt very much as though we're not trusted to get on with things and that's been uncomfortable at times. (Participant 5, VCS, Yr2)

### Culture

There was a consistent view that each collaborative partner brought different cultural norms and practices which often were contrasting. Some of these differences surrounded timeframes for the delivery of activities relating to the collaboration and others on more practical issues, such as differences in remuneration and annual leave allowance. In terms of the former, each of the different sectors represented commented on how other organizations worked at differing speeds. Academia was often perceived to take too much time to deliver and that this caused difficulties in the local authority where often insight and findings were required quickly for decision-making:

I think there is a different language between academia and the local authority, and the third sector and I think critically, there's often a difference in time scales, so I think academia, all the wheels turn quite slowly, and as I was saying it, you can perfectly well accept, can't you, that you start a research project and it might take two or three years and then you see some results at the end of it. Local authorities, I think, are used to and want to make decisions potentially quite quickly… ‘can you tell us about the research evidence relating to this particular decision, and it is going to cabinet in six weeks’ time’. (Participant 1, local authority, Yr1)

Interestingly though, as the collaboration developed there were minimal tensions between the local authority and academic partners in relation to the pace of work or the interactions with other partners:

I think the council's worked exceptionally well with our academic partners. (Participant 1, local authority, Yr2)

The pace of delivery for the VCS however was suggested to be in stark contrast with a propensity to deliver quickly and achieve results. This, perhaps, is due to funding stipulations in the third sector traditionally being short-term and premised on expedient delivery and outcome success:

In the third sector, partners at the moment seem to want to rush and get everything done yesterday because that's what they are used to….’Now get things done!’ so you know, it is different ways of working, and how people, well what people are used to, which I guess relates to culture and practice. (Participant 2, local authority, Yr1)

A number of participants recognized that these differences in cultural norms could be potentially problematic as the collaboration matured:

I think that a few tensions and conflicts are bubbling because people work differently, but I wouldn't put it as strong as conflict because I think we're still working it through. But we need to be careful that they don't become bigger issues than they need to be if that makes sense. (Participant 4, local authority, Yr1)

An open culture of dialogue and sharing concerns between partners worked effectively in Year 2 to enable cultural difference to be recognized, discussed and negotiated. However, time to engage in these sorts of conversations were often overshadowed by a mandate to deliver on actions and prioritize the outcomes of the work. This reflected a tension between the need for measurable progress (e.g. outputs, deliverables, timelines) and the relational labour required to sustain equitable, effective partnerships. This was considered a risk:

There's potential risks of having more transactional type of interactions…perhaps you would be better off to spend a little bit of time less formally interacting as a group of people which might then actually make the day-to-day engagement more straightforward because you've built a bit a bit more of a rapport. (Participant 17, Academia, Yr2)

### Working within political structures

Despite the initial perception that all collaborators had equal power, the local authority were ultimately the lead organization for the HDRC. This meant that other organizations needed to operate and understand the political nuances of local government and conform to the restrictions and limitations of working in this environment:

The difficulty we have got as a local authority are the restrictions and we are a politically guided organisation, whereas universities and other partner organisations…they have not got all that, I'm not going to say bureaucracy, but they've not got all those restrictions. (Participant 2, local authority, Yr1)

All organizations had elements of internal-flux, but with a focus on tackling health inequalities through local government decision-making it was clear that policy-cycles and political timing was going to be an issue to consider for the collaboration. A dynamic that was largely outside of the control of any of the collaboration organizations and steered by wider national and regional contexts. This both offered clear opportunities for advancing policy decisions, but also some constraints in managing political turnover and electoral successions:

There will be at least two local elections during the time of this project, which means the elected members changing and people’s recollections changing and things. So, I think that could be a challenge as well. I think that could be a really frustrating component of it as well. We'll be getting the elected members on board keeping them on board and getting them to appointment at the end of the project. They are using evidence and research in ways that they weren’t doing previously. (Participant 6, Academia, Yr1)

### Aligning visions and outcome expectations

The longitudinal data demonstrated a cohesive vision of the collaboration from all associated members and, in short, that was to improve lives for individuals and communities in the area. Nonetheless, this was viewed through a slightly different lens for each of the stakeholder groups.

The VCS partners described the ability to eventually have programmes to deliver in communities that were more finely attuned to people’s wants and needs and to continue to establish a firm and long-standing partnership with the local authority:

Our aspirations are that we will get good quality information to help us deliver services that support people…which will then obviously help them in the long term in terms of health, wellbeing, their own aspirations. *(Participant 4, VCS, Yr1)*

Local authority partners emphasized long-term sustainability of research funding and the increased capacity and capability of their workforce to utilize research—this would also extend to elected decision-makers who, in turn, would make better evidence-informed decisions about the population they serve:

I'd like us to see that we are generating our own research, where there are gaps, and we are using the research that other people are doing. We're feeding that into our decision-making processes and because of that, we're making better decisions and things are changing, or at least we have the potential for change. So on a very, very simple two-sentence level, we are getting the evidence, we're feeding the evidence into decision-making processes and that is then translating into better decisions, policies and action. (Participant 1, local authority, Yr1)

Academic partners stressed the importance of reconfiguring perceptions of research in the local authority and particularly with VCS organizations. With the expectation that research was not to be ‘feared’ or seen as something that is exclusively done by those in academia. Academics also noted the need to publish and disseminate research to show academic and societal impact.

## Discussion

Tackling health inequalities is complex and requires a wide-range of stakeholders working across multiple sectors to influence change (Woodall and Cross 2021). Working in these ways, however, is challenging and many collaborations fail to achieve the desired outcomes. This paper sought to understand longitudinal process issues in a collaboration seeking to reduce health inequalities in a geographical region in the UK—such a contribution can inform future interventions and learning to enhance the likelihood of success. This study gathered the experiences of participants over a 2-year period; these participants were key constituents in the conception and delivery of an HDRC in the UK—a programme which is centred around local government research capacity and development to understand health determinants and to improve health outcomes in communities (Hampshaw *et al*. 2024). Drawing on a range of stakeholders from different sectors in the collaboration, the study used longitudinal qualitative approaches to explore expectations of working collaboratively and how this materialized in practice.

Partnerships and collaborations have been seen as a panacea for tackling health inequalities and often regarded as providing a feel good factor through doing things differently (Dickinson and Glasby 2010). They are, however, difficult and can fail (Wildridge *et al*. 2004). While the formal governance structure of the collaboration positioned the local authority as the lead organization—holding financial responsibility and contractual accountability—participants initially anticipated a model of distributed power and shared decision-making. However, as the partnership matured, this assumption was challenged in practice. Despite the rhetorical commitment to equality among partners, several participants began to reflect on a clear power differential, particularly as the collaboration moved from vision-setting to operational decisions. This mirrors what collaborative governance literature has long observed in that despite formal frameworks, power often concentrates around organizations with status, budgetary control, and bureaucratic legitimacy (Ansell and Gash 2008). Our findings suggest that power was not monolithic but multifaceted; e.g. financial power linked to control of budgets, policy power through influence over strategic priorities, political power tied to elected authority, etc. Further exploration is warranted within the collaboration, and others, to determine whether these dynamics operate differently across specific areas of work or whether it is an overarching zero-sum power relationship.

While there was no evidence of failure in the collaboration under study; there were clear cultural differences between organizations which had the potential to manifest and hinder productive working. Differences in approach could be demonstrated in very practical ways—differences in annual leave entitlement amongst key individuals—but also in the pace of delivery and decision-making. On the latter point, the VCS was regarded as wanting to work more expediently than other partners and deliver early. This is unsurprising given the general trend for third-sector providers to be commissioned on short-term funding arrangements and achieving objective measures of success (Harradine and Greenhalgh 2012). While some of these issues had begun to be apparent, it was clear that they had not proved problematic. That said, other HDRC collaborations have suggested the necessity to align expectations and address cultural variances in the first year of the partnership; with this work considered a delicate process (Newbury-Birch *et al*. 2024). In terms of cultural difference, small and practical issues matter (Dickinson and Glasby 2010). Differences in discourse and working conditions are rarely discussed but can potentially manifest into challenging conversations if left unaddressed (Mason *et al*. 2015). Shared language and understanding, in contrast, has been observed to increase relational trust between partners (Such *et al*. 2022). The benefits of the HDRC programme being relatively long-term in nature (5-years) suggests that these shared understandings can be achieved and it is apparent from other studies that issues can be reconciled within the timeframe (Holding *et al*. 2024). That said, theory suggests that some organizations do not often have the time or energy to invest in time-intensive collaborative processes (Ansell and Gash 2008).

The central understanding that the collaboration was seeking to address health inequalities was firmly understood by all participants and this shared vision offered a useful anchor for establishing shared comprehension of the work both in the formative period of the collaboration and in the delivery of activities. The notion that the collaboration offered distinct benefits, with each organization compensating for each other’s limitations was also apparent in the data. Other HDRCs have reported how learning from strengths within the team is critical in forming a strong partnership and understanding (Holding *et al*. 2024). Recognizing individual and organizational assets within collaborations seems an important first-stage in any collaborative endeavour. There is little empirical evidence currently about the VCS and their partnerships and relationships with other sectors in relation to addressing health inequalities, but in this study the VCS were regarded as an antidote to the more rigid and often bureaucratic statutory sector and seen as a trusted conduit to facilitate community access. Such characterizations of the VCS are not unusual in supporting the delivery of health interventions to disadvantaged communities (Bach-Mortensen *et al*. 2018). Notwithstanding, the VCS themselves recognized limitations in capability and capacity for research and in identifying and implementing evidence-based interventions (Bach-Mortensen *et al*. 2018).

Mistrust was an impediment to successful collaborative working, exemplified through some participants feeling that they were often micromanaged and not enabled to deliver fully. Much of this was not rooted in current dynamics, but stemmed from prior organizational histories between organizations and deep-rooted feelings influenced by previous working relationships. Collaborative governance theory highlights how historic tensions and challenges between partners reduces trust (Ansell and Gash 2008). Indeed, a whole myriad of studies suggest that trust lubricates successful collaborative working and how partnerships are more likely to succeed if trust is present (Alderwick *et al*. 2021). Trust enables risk-taking, fosters open communication and supports distributed leadership, all of which are critical for effective inter-organizational working (Ansell and Gash 2008). Systematic review evidence also suggests that historical working relationships do matter and can often negate effective working:

Historic relationships between agencies—present or absent; good or bad—shaped how local partnerships developed and functioned. (Alderwick *et al*. 2021: 7)

Less functionality in exchanges between the constituents of the collaboration and more opportunities to lubricate personal and organizational dynamics was suggested as a potential antidote to moving beyond historical tensions. Theory suggests that face-to-face dialogue is a necessary but not sufficient condition for successful collaboration (Ansell and Gash 2008), but clearly eroding unhelpful and historic stereotypes of organizations (and individuals) seems a positive way of strengthening a collaborative.

Effective policy-making is one of the key resources in improving public health, reducing health inequalities and fostering supportive environments (van de Goor *et al*. 2017, Woodall and Cross 2021). However, policy decisions can frequently be underpinned by political timeliness (based on perceived short-term opportunities and political preferences) or mandates from central government, rather than credible research evidence (van de Goor *et al*. 2017). It was apparent that working in a political context would be essential for all of the collaborative partners given the focus on local government (Homer *et al*. 2022). The notion of political timeliness was key and how this could contest research processes which could be slower to respond to immediate need (Woodall *et al*. 2024). University partners were seen to offer rigour in research and evaluation processes, but some concerns were raised about the timeframe for achieving this which might contest the pace of delivery required by local government. This is a tension that is becoming relatively well-recognized with research often regarded as a ‘luxury’ in local government and not embedded in daily practice (Holding *et al*. 2024, Woodall *et al*. 2024).

The study provides some insight into processes of a collaboration working toward reducing health inequalities, but it is clear that such partnerships are dynamic and evolving. The longitudinal qualitative design offered distinct benefits in providing timely assessment of the collaboration’s working practices and in providing feedback to develop the cohesiveness of partners. The paper intends to offer perspectives that can be transferred to other partnerships; however, it is important to recognize the contextual specificity of the HDRC model and the UK-based context. The long-term funding (5 years) provided by the NIHR is an enabling feature that cannot be assumed in other contexts. Short-term funding models in other programmes might discourage the development of collaborative capacity, particularly where there is pressure for rapid results. Indeed, further work on the benefits of collaborative working for addressing the social determinants outside of the UK is warranted a key critique of the Ottawa Charter was that it reflects a predominantly Western, industrialized perspective, which inadvertently marginalizes other global communities. The discourse underpinning its development has been criticized for reinforcing power imbalances and privileging Western-centric worldviews, effectively silencing non-Western voices in the process (Nutbeam 2008, McPhail-Bell *et al*. 2013). While this study offers transferability, it much be exercised with caution.

## Conclusions

The Ottawa Charter’s vision of health promotion as a collective, systems-level endeavour is clearly reflected in the HDRC model but little is understood about which collaborations work, for whom and in what contexts (Alderwick *et al*. 2021). This paper sought to explore the views of constituents working in a multi-sector collaboration seeking to address health inequalities using a longitudinal design to track changes over time. What distinguishes this study from existing partnership literature is its dual focus: first, on the HDRC as a novel national initiative embedding research capacity within local government; and second, on revisiting the Ottawa Charter’s principles through a contemporary lens. By using a longitudinal design to examine how these principles play out in practice, the paper offers unique insights into the operational realities of intersectoral collaboration beyond traditional health and social care partnerships.

Subscribing to a ‘shared vision’ is a long-standing trope in collaborative working and one which is indeed critical for success (Cross and Woodall 2024). While all partners had a slight difference in their ‘road map’ for success the shared endpoint was apparent and clear—to reduce health inequalities. Partners had distinct strengths which, when brought together, provided an holistic package that had the potential to effectively navigate the complex challenges posed by entrenched health inequalities. That said, tensions could potentially emanate between the constituents due to differing ways of working and accepted cultural norms. It is not unusual though for these tensions to be apparent in collaborative working, especially when bringing diverse sectors together (Holding *et al*. 2024).

There is a danger of ‘partnership working as a panacea and imbuing it with overly ambitious aspirations’ (Dickinson and Glasby 2010: 821). The findings caution against viewing partnership as a universal remedy and instead underscore the need to focus on the collaborative process as well as tangible outcomes. These findings have clear implications for both policy and practice. For health collaborations, the study underscores the need to embed collaborative governance principles into partnership arrangements, ensuring clarity on roles; power-sharing; and decision-making processes. For practitioners across all sectors—including local authorities; academia; and the voluntary and community sector, the data suggest that building trust, aligning cultural norms, and investing time in relationship management are as important as technical expertise for achieving health equity. Revisiting and potentially reconfiguring the Ottawa Charter may be necessary to ensure its principles remain relevant and responsive given contemporary challenges and issues with intersectoral collaboration to address health inequalities. Future research should continue to explore how such partnerships evolve over time, how trust can be built and sustained across sectors and under what conditions intersectoral collaboration truly leads to measurable improvements in health equity.

## Data Availability

Data available on request from the corresponding author.

## References

[daaf234-B1] Adeoye-Olatunde OA, Olenik NL. Research and scholarly methods: semi-structured interviews. J Am Coll Clin Pharm 2021;4:1358–67. 10.1002/jac5.1441

[daaf234-B2] Alderwick H, Hutchings A, Briggs A et al The impacts of collaboration between local health care and non-health care organizations and factors shaping how they work: a systematic review of reviews. BMC Public Health 2021;21:1–16. 10.1186/s12889-021-10630-1 33874927 PMC8054696

[daaf234-B3] Ansell C, Gash A. Collaborative governance in theory and practice. J Public Adm Res Theory 2008;18:543–71. 10.1093/jopart/mum032

[daaf234-B4] Armistead C, Pettigrew P, Aves S. Exploring leadership in multi-sectoral partnerships. Leadership 2007;3:211–30. 10.1177/1742715007076214

[daaf234-B5] Bach-Mortensen AM, Lange BCL, Montgomery P. Barriers and facilitators to implementing evidence-based interventions among third sector organisations: a systematic review. Implement Sci 2018;13:103. 10.1186/s13012-018-0789-7 30060744 PMC6065156

[daaf234-B6] Boydell L . Partnership Framework: a Model for Partnerships in Health. Dublin: The Institute of Public Health in Ireland, 2001.

[daaf234-B7] Braun V, Clarke V. Successful Qualitative Research. London: Sage, 2013.

[daaf234-B8] Braun V, Clarke V. A critical review of the reporting of reflexive thematic analysis in Health Promotion International. Health Promot Int 2024;39:1–12. 10.1093/heapro/daae049 PMC11132294 38805676

[daaf234-B9] Corbin JH, Jones J, Barry MM. What makes intersectoral partnerships for health promotion work? A review of the international literature. Health Promot Int 2018;33:4–26. 10.1093/heapro/daw061 27506627 PMC5914378

[daaf234-B10] Cross R, Woodall J. Green & Tones’ Health Promotion: Planning & Strategies. London: Sage, 2024.

[daaf234-B11] Dickinson H, Glasby J. Why partnership working doesn't work. Public Manag Rev 2010;12:811–28. 10.1080/14719037.2010.488861

[daaf234-B12] Evans D, Killoran A. Tackling health inequalities through partnership working: learning from a realistic evaluation. Crit Public Health 2000;10:125–40. 10.1080/09581590050075899

[daaf234-B13] Fransham M, Herbertson M, Pop M et al Level best? The levelling up agenda and UK regional inequality. Reg Stud 2023;57:2339–52. 10.1080/00343404.2022.2159356

[daaf234-B14] Hampshaw S, Morling J, Black M. Investing in research infrastructure to address health inequalities: learning by doing. Public Health in Practice 2024;7:100460. 10.1016/j.puhip.2023.100460 38235263 PMC10792754

[daaf234-B15] Harradine D, Greenhalgh K. LinkAge plus: lessons for third sector organisations and commissioners. Int J Public Sect Manag 2012;25:391–403. 10.1108/09513551211252404

[daaf234-B16] Holding E, Gettings R, Foster A et al Developing the embedded researcher role: learning from the first year of the National Institute for Health and Care Research (NIHR), Health Determinants Research Collaboration (HDRC), Doncaster, UK. Public Health in Practice 2024;7:100516. 10.1016/j.puhip.2024.100516 38846108 PMC11153224

[daaf234-B17] Holt DH, Aveling E-L. Achieving partnership synergy: resource inputs, shared mission and interdependencies in Danish health promotion partnerships. Health Promot Int 2023;38:daac203. 10.1093/heapro/daac203 36795098

[daaf234-B18] Homer C, Woodall J, Freeman C et al Changing the culture: a qualitative study exploring research capacity in local government. BMC Public Health 2022;22:1341. 10.1186/s12889-022-13758-w 35836209 PMC9281003

[daaf234-B19] Hubley J, Copeman J, Woodall J. Practical Health Promotion. Cambridge: Polity Press, 2021.

[daaf234-B20] Jones J, Barry MM. Exploring the relationship between synergy and partnership functioning factors in health promotion partnerships. Health Promot Int 2011;26:408–20. 10.1093/heapro/dar002 21330307

[daaf234-B21] Judge K, Bauld L. Learning from policy failure? Health action zones in England. Eur J Public Health 2006;16:341–3. 10.1093/eurpub/ckl068 16698883

[daaf234-B22] Lindström B . Workshop salutogenesis and the future of health promotion and public health. Scand J Public Health 2018;46:94–8. 10.1177/1403494817743902 29552971

[daaf234-B23] Marmot M, Allen J, Boyce T et al Health Equity in England: the Marmot Review 10 Years on. London: Institute of Health Equity, 2020. 10.1136/bmj.m693 32094110

[daaf234-B24] Mason A, Goddard M, Weatherly H et al Integrating funds for health and social care: an evidence review. J Health Serv Res Policy 2015;20:177–88. 10.1177/1355819614566832 25595287 PMC4469543

[daaf234-B25] McGill E, Marks D, Er V et al Qualitative process evaluation from a complex systems perspective: a systematic review and framework for public health evaluators. PLoS Med 2020;17:e1003368. 10.1371/journal.pmed.1003368 33137099 PMC7605618

[daaf234-B26] McPhail-Bell K, Fredericks B, Brough M. Beyond the accolades: a postcolonial critique of the foundations of the Ottawa Charter. Glob Health Promot 2013;20:22–9. 10.1177/1757975913490427 23797937

[daaf234-B27] Newbury-Birch D, Harbin K, Adamson A et al Establishing Research Ecosystems in Local Government: Ten Lessons from the Front Line of the First Year of the NIHR Health Determinants Research Collaborations (HDRCs). London: NIHR Open Research, 2024.

[daaf234-B28] NIHR . Health Determinants Research Collaborations, 2024.https://www.nihr.ac.uk/about-us/what-we-do/working-with-partners/local-authorities/health-determinants-research-collaborations (14 July 2025 date last accessed).

[daaf234-B29] Nutbeam D . What would the Ottawa charter look like if it were written today? Crit Public Health 2008;18:435–41. 10.1080/09581590802551208

[daaf234-B30] Patton MQ . Qualitative Research & Evaluation Methods: Integrating Theory and Practice. London: Sage, 2014.

[daaf234-B31] Perkins N, Hunter DJ, Visram S et al Partnership or insanity: why do health partnerships do the same thing over and over again and expect a different result? J Health Serv Res Policy 2020;25:41–8. 10.1177/1355819619858374 31461362

[daaf234-B32] Smith K, Bambra C, Joyce K et al Partners in health? A systematic review of the impact of organizational partnerships on public health outcomes in England between 1997 and 2008. J Public Health 2009;31:210–21. 10.1093/pubmed/fdp002 19182048

[daaf234-B33] South J, Woodall J, Stansfield J et al A qualitative synthesis of practice-based learning from case studies on COVID community champion programmes in England, UK. BMC Public Health 2024;24:7. 10.1186/s12889-023-17470-1 38166766 PMC10759547

[daaf234-B34] Such E, Smith K, Woods HB et al Governance of intersectoral collaborations for population health and to reduce health inequalities in high-income countries: a complexity-informed systematic review. Int J Health Policy Manag 2022;11:2780. 10.34172/ijhpm.2022.6550 35219286 PMC10105187

[daaf234-B35] Thomas SL, Kickbusch I, Kökény M et al 40 years of the Ottawa charter for health promotion—reaffirming health for all. Health Promot Int 2025;40:1–3. 10.1093/heapro/daaf143 40836494

[daaf234-B36] van de Goor I, Hämäläinen R-M, Syed A et al Determinants of evidence use in public health policy making: results from a study across six EU countries. Health Policy 2017;121:273–81. 10.1016/j.healthpol.2017.01.003 28139253 PMC5754321

[daaf234-B37] WHO . Ottawa Charter for health promotion. Health Promot 1986;1:405–v. 10.1093/heapro/1.4.405

[daaf234-B38] WHO . Report of a Conference on Intersectoral Action for Health: A Cornerstone for Health-for-all in the Twenty-First Century, 20–23 April 1997, Halifax, Nova Scotia, Canada. Geneva: World Health Organization, 1997.

[daaf234-B39] Wildridge V, Childs S, Cawthra L et al How to create successful partnerships—a review of the literature. Health Info Libr J 2004;21:3–19. 10.1111/j.1740-3324.2004.00497.x 15186286

[daaf234-B40] Willis C, Greene J, Abramowicz A et al Strengthening the evidence and action on multi-sectoral partnerships in public health: an action research initiative. Health Promot Chronic Dis Prev Can 2016;36:101–11. 10.24095/hpcdp.36.6.01 27284702 PMC4910446

[daaf234-B41] Woodall J, Cross R. Essentials of Health Promotion. London: Sage, 2021.

[daaf234-B42] Woodall J, Freeman C. Where have we been and where are we going? The state of contemporary health promotion. Health Educ J 2020;79:621–32. 10.1177/0017896919899970

[daaf234-B43] Woodall J, Homer C, South J et al Evidence-based decision making in a climate of political expediency: insights from local government. Perspect Public Health 2024. 10.1177/17579139241256879 PMC13213032 38859638

[daaf234-B44] Woodall J, Morley L. Health promotion: reconfiguring nurses’ practice to reduce social inequalities. Nursing Standard 2024;39:47–50. 10.7748/ns.2024.e12266 38404065

